# Epithelial PD‐L1 expression at tumor front predicts overall survival in a cohort of oral squamous cell carcinomas from Sudan

**DOI:** 10.1002/cre2.666

**Published:** 2022-09-30

**Authors:** Nuha M. Gaafar, Tarig Al‐Hadi Osman, Mariam Elsheikh, Israa Abdulrahman Ahmed, Harsh Dongre, Siren Fromreide, Ahmed M. Suleiman, Anne C. Johannessen, Elisabeth S. Nginamau, Daniela‐Elena Costea

**Affiliations:** ^1^ The Gade Laboratory for Pathology and Centre for Cancer Biomarkers (CCBIO), Department of Clinical Medicine, Faculty of Medicine University of Bergen Bergen Norway; ^2^ Centre for International Health, Department of Global Public Health and Primary Care, Faculty of Medicine University of Bergen Bergen Norway; ^3^ Department of Oral and Maxillofacial Surgery, Faculty of Dentistry University of Khartoum Khartoum Sudan; ^4^ Department of Pathology Haukeland University Hospital Bergen Norway; ^5^ Khartoum Dental Teaching Hospital Khartoum Sudan

**Keywords:** biomarker, invasive tumor front (ITF), oral squamous cell carcinoma (OSCC), overall survival (OS)

## Abstract

**Background:**

We recently described the tumor immune microenvironment (TIME) in oral squamous cell carcinomas (OSCC) from Sudan by assessing the core of the lesions. However, the invasive tumor front (ITF) is the most active part of OSCC lesions; thus, TIME should also be characterized at the ITF in this patient cohort.

**Objectives:**

We aimed to evaluate patterns of immune cell infiltration at the ITF in a cohort of OSCC patients from Sudan previously investigated at the tumor center and their association with clinicopathological parameters.

**Methods:**

This study was performed on a prospective cohort of 22 OSCC patients attending Khartoum Dental Teaching Hospital with a median follow‐up of 48 months. Inflammatory infiltrate densities of CD4‐, CD8‐, FoxP3‐, CD20‐, CD66b‐, M1 (CD80/CD68)‐, M2 (CD163/CD68)‐, and PD‐L1‐positive cells were assessed at the ITF by immunohistochemistry, followed by digital quantitative analysis at the stromal and epithelial compartments separately. Histopathological parameters such as the worst pattern of invasion, differentiation, and tumor budding (TB) were also assessed. Correlations between clinicopathological parameters and survival analysis were investigated using SPSS.

**Results:**

All inflammatory cell subsets investigated were found to be higher in the stromal compartment as compared to the epithelial one, except for the PD‐L1^+^ subset. Stromal infiltration with the CD8^+^ cell subset was associated with low TB. Kaplan–Meier analyses identified higher epithelial and stromal CD4^+^ cell subsets. The presence of PD‐L1 was found to be associated with unfavorable overall survival. Further, Cox's regression analysis using an age‐ and tumor‐stage‐adjusted model identified epithelial PD‐L1 expression at the ITF as the only independent prognosticator.

**Conclusions:**

Epithelial PD‐L1 expression at the ITF was found to be an independent prognostic biomarker for OSCC in a cohort of Sudanese patients.

## 1 INTRODUCTION

Oral cancer accounts for approximately 4% of all malignancies (Omura, 2014). More than 90% of oral cancers are squamous cell carcinomas (OSCC) (Montero & Patel, 2015). The most important OSCC risk factors, which are tobacco (both smokd and smokeless) and alcohol, have a synergistic effect if used together (Chaturvedi et al., 2013). The prognosis is highly heterogeneous, with an overall 5‐year survival rate of approximately 50% (Khan et al., 2020). OSCC management is mainly surgical by attempting complete resection with adjuvant radio‐ and chemotherapy (Montero & Patel, 2015). In advanced cases of OSCC, modulating therapies, for example, with PD‐1‐blocking antibodies showed promising results (Harrington et al., 2017). This indicates that the tumor immune microenvironment (TIME) is a significant regulator of tumor progression and thus brings tumor immunology to the front line of OSCC research (Binnewies et al., 2018).

TIME is rich and complex, containing heterogeneous cell subsets (Binnewies et al., 2018). The immune cells that infiltrate cancer lesions might play different roles in various cancer types and even in different areas of the same tumor (Fridman et al., 2017). Currently, there is solid evidence that TIME is of major importance for OSCC invasion and progression (Hadler‐Olsen & Wirsing, 2019). Tumor‐infiltrating immune cells are frequently observed in OSCC lesions, which are highly immunogenic tumors (Ferris, 2015). A high tumor inflammatory response in the stroma has been identified to be an independent predictor for better disease‐specific survival (DSS) when analyzed separately in human papillomavirus (HPV)‐positive and HPV‐negative oropharyngeal squamous cell carcinoma patients (Haave et al., 2018). In a meta‐analysis of studies investigating T‐cell subtypes, high levels of infiltrating CD3^+^ T cells (pan T‐cell marker), CD4^+^ T‐helper cells, or CD8^+^ T cytotoxic cells were found to be associated with longer survival, whereas high numbers of FoxP3^+^ T‐regulatory cells were found to be associated with decreased survival (Hadler‐Olsen & Wirsing, 2019). The same meta‐analysis identified two out of four analyzed studies that showed a significant association of high tumor infiltrates of B lymphocytes (recognized by the biomarkers CD19 and CD20) with better survival (Hadler‐Olsen & Wirsing, 2019). Many studies have also focused on macrophages in OSCC, and the majority pointed toward an association with poor survival of this cell type (Ai et al., 2021; Alves et al., 2018; Kalogirou et al., 2021; Ni et al., 2015). A meta‐analysis on tumor‐infiltrating macrophages has shown a significant negative effect on overall survival (OS) in the presence of a high number of infiltrating CD163^+^ subtype of macrophages (traditionally indicated as the M2, protumor subtype of macrophages) (Hadler‐Olsen & Wirsing, 2019). The amount and subset distribution of the tumor inflammatory infiltrate is not the sole determinant of the immunologic tumor–host interaction (Kalogirou et al., 2021). The consequences of the immunologic host reaction are also regulated by immune‐escape mechanisms that tumors might use to escape the antitumor effect of the inflammatory infiltrate; one of these is the expression of PD‐L1 (Spranger, 2016).

Quantification of the immune cell infiltrates, particularly at the invasive tumor front (ITF, referred to as the tumor–stromal interface including up to six tumor cell layers or isolated single tumor cells or small islands at the outmost border of a cancer lesion), for prognostication in OSCC has been pinpointed for almost 30 years (Bryne et al., 1992). However, it took a while till TIME was investigated separately at the ITF and tumor center (TC), and interestingly, a very recent study, comparing ITF and TC, showed that the intratumoral spatial arrangement of inflammatory infiltrates and the expression of immune markers was homogeneous in these two areas in the majority of OSCC cases (Boxberg et al., 2019). On the contrary, a study on breast cancer demonstrated that tumor‐infiltrating lymphocytes (TILs) and TIL subtypes showed significantly differential distribution between the two tumor areas (König et al., 2019). In colorectal cancer, it was also demonstrated that CD3^+^ and CD8^+^ lymphocytes were more abundant at the ITF than at the TC and that their amount and arrangement were associated with OS (Galon et al., 2006). We previously showed that despite the high abundance of inflammatory cells in tumor‐associated stroma of the TC, a low intraepithelial infiltration with immune cells was observed at the TC of the majority of investigated OSCC lesions (Gaafar et al., 2022). When an immunoscore was created based on the combination of individual CD4 and FoxP3 intraepithelial TC scores, this score was found to be correlated to tumor progression (Gaafar et al., 2022). However, a more precise prognostic value for different components of TIME was not identified at the TC. Therefore, considering that ITF was proven as the most active part of OSCC lesions, this study aimed at further evaluation of the patterns of immune cell infiltration at the ITF in the same cohort of OSCC patients from Sudan and their association with clinicopathological parameters.

## 2 MATERIALS AND METHODS

### 2.1 Patient cohort

Twenty‐two OSCC patients recruited during 2014–2015 at the Khartoum Dental Teaching Hospital, Sudan, were included in this study. The participants were also part of the prospective cohort in which TIME in the TC was previously studied (Gaafar et al., 2022). Ethical approval was obtained from the National Health Research Ethics Committee, Federal Ministry of Health, Sudan (FMOH/RD/SEC/09), and the Regional Ethical Committee in Norway (REKVest 3.2006.2620 REKVest 3.2006.1341). Briefly, the inclusion criteria for OSCC were age >18 years, histopathologically confirmed primary p16‐negative OSCC, no previous cancer‐specific treatment, and having given written consent. The exclusion criteria included patients who were critically ill and positive for human immunodeficiency virus and/or hepatitis B surface antigen. Clinical and demographic information was obtained from the patient's hospital records. In addition, a routine dental examination recording missed teeth (MT) and decayed teeth (DT), and the community periodontal index of treatment needs (CPITNs) was performed on participating individuals by four dentists who were trained and calibrated before data collection. Tumor node metastasis staging and tobacco consumption were recorded as previously described (Gaafar et al., 2022). OS was defined as the time (in months) from the date of diagnosis till the end date of the study. The current study investigated only the primary OSCCs that had available formalin‐fixed, paraffin‐embedded blocks containing ITF. Therefore, 14 cases from our previously analyzed cohort at the TC (Gaafar et al., 2022) could not be analyzed in the current study due to the absence of the ITF in the tissue blocks. The ITF was defined as a band of 100 µm tissue area including the outermost invasive tumor islands and the surrounding stroma. Tumor budding (TB), defined as an island of less than five cancer cells (including single cells only as one bud), was evaluated as previously described (Almangush et al., 2014). The worst pattern of invasion (WPI) was assessed as reported by Brandwein‐Gensler et al. (2005).

### 2.2 Immunohistochemistry (IHC)

Single‐ and double‐staining IHC were performed as previously described (Gaafar et al., 2022). Deparaffinization and rehydration of tissue sections were performed by using xylene and graded alcohol concentrations (100%, 96%, and 70%), respectively. A microwave oven (Whirlpool, Benton Harbor, MI, USA) was used for epitope retrieval for 25 min in total, first at 950 W for 7–8 min until the retrieval solution boiled and then at 350 W for 17–18 min. Two different in‐house retrieval solutions were used: citrate (2.1 g citric acid [VWR International, Radnor, PA, USA], 9 ml of 3 N NaOH [Sigma‐Aldrich, St. Louis, MO, USA], 1 L milliQ water, pH 6.0) and Tris/EDTA (1.205 g Tris‐base [Sigma‐Aldrich], 0.395g EDTA [VWR International], 1 L milliQ water, pH 9.0). Blockage of the unspecific binding sites with 10% normal goat serum (Agilent, Santa Clara, CA, USA) and 3% bovine serum albumin (Agilent) in Tris‐buffered saline (Sigma‐Aldrich) was then performed. After primary antibody incubation for the detection of CD4, CD8, CD20, CD66b, FoxP3, PD‐L1, and PanCK antigens, the EnVision™ Visualization System (Agilent Technologies) was used for all antibodies, except for PanCK, where MACH3 (Biocare Medical, Pacheco, CA, USA) was used. Overnight incubation with the first primary antibodies (CD80 or CD163) at 4°C was performed for double staining. Visualization of the first primary antibody was then performed using 3, 3′‐diaminobenzidine (DAB) chromogen and a heat‐induced denaturation step was performed before incubation with the second primary antibody (CD68) for 1 h at room temperature. EnVision™ Double Stain System (Agilent Technologies) was used for the remaining steps. After blocking the reactions from the primary antibodies and the indigenous tissue alkaline phosphatase (AP), visualization of the second primary antibody was performed by incubation with labelled AP anti‐mouse antibody and the liquid permanent Red (LPR) substrate chromogen. Aperio Scanscope® CS Slide Scanner was used to scan (×40 magnification) the immuno‐stained slides and ImageScope (Aperio Technologies Inc., Vista, CA, USA) was used for annotations and quantification. Manual annotations separately for stromal and epithelial compartments were performed at the ITF (from 5 to 7 regions with inflammatory infiltrate hotspots, with a total analyzed area of a minimum of 0.2 mm^2^) using quantifying algorithms previously established in our laboratory (Gaafar et al., 2022).

### 2.3 Statistics

Evaluation of CD4, FoxP3, CD20, and PD‐L1 immunomarkers was presented as the absolute number of identified positive cells divided by the total area that was analyzed. Evaluation of CD8 and CD66b immunomarkers was presented as the percentage of the total stained area within the total analyzed area. Evaluation of M1 and M2 macrophage subtypes was presented as the percentage of positive DAB pixels divided by the percentage of LPR‐positive pixels within the total analyzed area. SPSS 25.0 software (SPSS, Chicago, IL, USA) was used for statistical analysis. The normal distribution of all data was explored using the Shapiro–Wilk test (parametric or nonparametric tests). *T*‐test was used for parameters/biomarkers with normal distribution, while skewed parameters/biomarkers were analyzed using the Mann–Whitney *U*‐test. For CD4, CD8, and CD20 markers, paired *t*‐test was used to compare their expression in stromal and epithelial compartments. For FoxP3, CD66b, and PD‐L1 markers and M1 and M2 subtypes, Wilcoxon's signed‐rank test was performed for the same reason. Statistical significance was considered when *p* < .05 (two‐sided). Each variable representing the type of immune cells was dichotomized into high and low groups by the median value for the whole cohort. Relationships between clinicopathological parameters and biomarkers and among different immune subsets in stromal and intraepithelial locations were assessed using the *χ* ^2^ test. Kaplan–Meier with log‐rank test was used for OS analysis, followed by multivariate Cox regression modelling to investigate the prognostic value of the studied biomarkers.

## 3 RESULTS

### 3.1 Cohort description

The patient's age ranged from 25 to 83 years (mean: 62.18 ± 12.41 years and median: 62.50 years). Males comprised 72.7%; poor oral hygiene was observed in 40.9% of patients; 40.9% of patients self‐reported a history of smoking, 50% self‐reported toombak use, and 22.7% self‐reported alcohol consumption. OSCC lesions were located mainly in the sulcular, labial, and buccal mucosa (54.5%), which are typical snuff dipping sites. Half of the patients presented at Stage IV with large tumors. About three‐quarters (72.8%) of the patients presented with lymph node involvement. Well‐differentiated tumors composed 36.4% of OSCCs, 81.8% showed Type 4 WPI, 54.5% presented with high TB (i.e., more than five buds), and 50.0% showed moderate lymphocytic infiltration. Patient demographics and tumor histopathological characteristics are shown in Table 1.

**Table 1:** Clinical features and histopathological characteristics of OSCC cases

Clinical features	OSCC (*n* = 22), *N* (%)
Age	
≥65 years	11 (50.0)
<65 years	11 (50.0)
Gender	
Male	16 (72.7)
Female	6 (27.3)
Diabetes	
Yes	3 (13.6)
No	19 (86.4)
Systemic disease	
Yes	4 (18.2)
No	18 (81.8)
Smoking	
Yes (past and/or current smoker)	9 (40.9)
No	13 (59.1)
Toombak	
Yes (past and/or current user)	11 (50.0)
No	11 (50.0)
Alcohol	
Yes (past and/or current user)	5 (22.7)
No	15 (68.2)
Missed teeth (MT) according to the population median	
Low	10 (45.5)
High	11 (50.0)
Decayed teeth (DT) according to the population median	
Low	13 (59.1)
High	8 (36.4)
Community periodontal index for treatment needs (CPITNs)	
1—CPI	3 (13.6)
2—CPI	7 (31.8)
3—CPI	7 (31.8)
Gingival index (GI)	
1—GI	3 (13.6)
2—GI	14 (63.6)
3—GI	1 (4.5)
Oral health index simplified (OHIS)	
1—OHIS	1 (4.5)
2—OHIS	8 (36.4)
3—OHIS	9 (40.9)
Clinical outcome	
Alive	9 (40.9)
Dead	13 (59.1)
Survival time	
Alive ≥24 months	9 (40.9)
Alive <24 months	13 (59.1)
Location	
Buccal, labial, and sulcular	12 (54.5)
Alveolar, palate, and retromolar	5 (22.7)
Tongue	2 (9.1)
Tumor size	
T1	2 (9.1)
T2	6 (27.3)
T3	4 (18.2)
T4	7 (31.8)
Lymph nodes involvement	
N0	2 (9.1)
N1	10 (45.5)
N2	6 (27.3)
N3	0 (0.0)
Tumor stage	
I	0 (0.0)
II	1 (4.5)
III	8 (36.4)
IV	11 (50.0)
Worst pattern of invasion (WPI)	
Type 1	2 (9.1)
Type 2	1 (4.5)
Type 3	0 (0.0)
Type 4	18 (81.8)
Type 5	1 (4.5)
Tumor budding (TB)	
Low budding (<5 buds)	9 (40.9)
High budding (≥5 buds)	13 (59.1)
Tumor grading	
Well‐differentiated	8 (36.4)
Moderately differentiated	10 (45.5)
Poorly differentiated	4 (18.2)
Lymphocytic infiltration	
Marked	5 (22.7)
Moderate	11 (50.0)
Little/none	6 (27.3)

### 3.2 Clinical correlations

**Figure 1:**
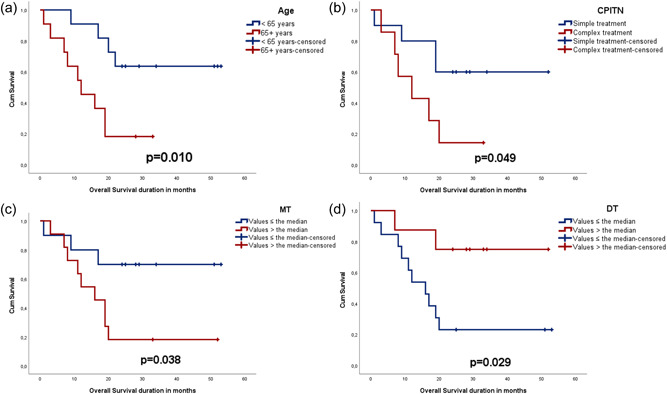
Kaplan–Meier curves depicting survival analysis and distribution of patients according to (a) age; (b) periodontal treatment needs (community periodontal index of treatment needs—CPITN); (c) missed teeth status (MT); and (d) decayed teeth status (DT) as predictors of overall survival.

Survival analysis showed that patients over 65 years (*p* = .010), with high MT (*p* = .038), with lower DT (*p* = .029), and who needed complex periodontal treatment (*p* = .049) had poor OS (Figure 1). In a multivariate model using all the variables above (Supporting Information: Table S1), older age (*p* = .037), and fewer decayed teeth (*p* = .010) were found to be associated with OS (*p* = .002).

### 3.3 Differential inflammatory cell infiltrate in the two tumor compartments

**Figure 2:**
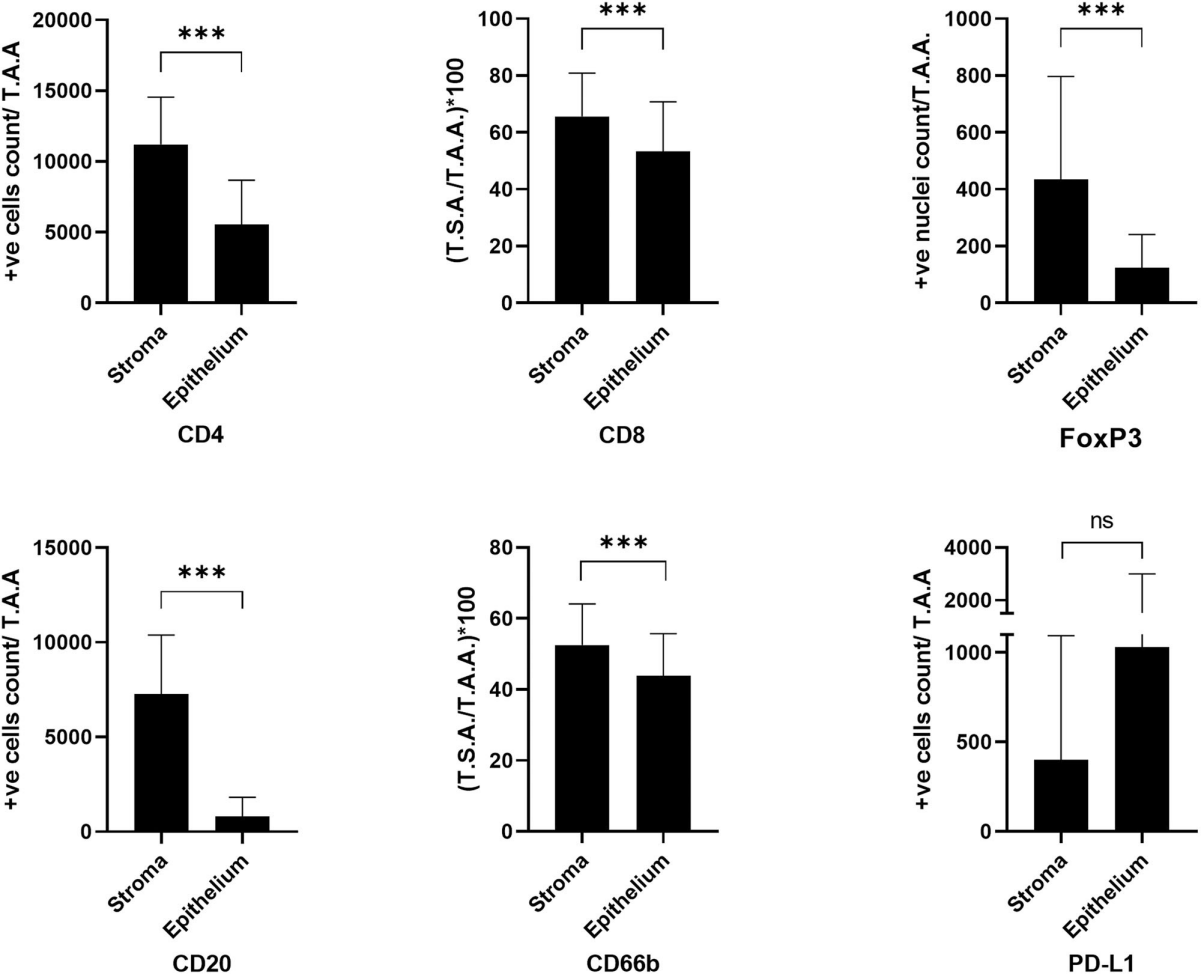
Bar graphs representing quantification of the inflammatory infiltrates in the two tumor compartments of the oral squamous cell carcinoma cases at the invasive tumor front. ***p < 0.001. NS, not significant; TAA, total analyzed area; TSA, total stained area.

Inflammatory cell infiltrates (all subsets, excluding PD‐L1^+^) were statistically significantly higher in the stroma than in the epithelial compartment of OSCC at the ITF (Supporting Information: Table S2). Only PD‐L1 had an opposite trend (not statistically significant) of higher expression in the epithelium than in the stromal compartment (Figure 2).

### 3.4 Correlation between immune cell subsets in the two tumor compartments

Immune cell infiltration of the CD4^+^ subset in the tumor‐associated stroma was positively (strong) correlated to the infiltration of the CD8^+^ cell subset. The presence of both CD4^+^ and CD8^+^ cell subsets was found to have a positive (strong) correlation with the presence of CD66b^+^ cell infiltrate. A positive (moderate) correlation was also observed between PD‐L1^+^ cells and the M2 phenotype of macrophages (Supporting Information: Table S3).

In the epithelial compartment, CD4^+^ cell infiltrates displayed a positive (moderate) correlation with CD20^+^ and PD‐L1^+^ cells. A positive (moderate) correlation between the number of PD‐L1^+^ cells and FoxP3^+^ cells was found. Infiltration of FoxP3^+^ T‐regulatory cells (T‐regs) negatively (moderate) correlated with infiltration of CD66b^+^ cells. Abundant CD66b^+^ cell infiltrates correlated with abundant CD8^+^ cell infiltrates in the epithelium (Supporting Information: Table S4).

The infiltrate amount of FoxP3^+^, CD20^+^, CD66b^+^, and PD‐L1^+^ cell subsets in the stromal compartment positively correlated with the respective infiltrates in the epithelial compartment at the ITF (phi = 0.90, *p* < .001; phi = 0.54, *p* = .013; phi = 0.43, *p* = .049 and phi = 0.61, *p* = .005, respectively).

### 3.5 Correlations between immune biomarkers and clinicopathological parameters

**Figure 3:**
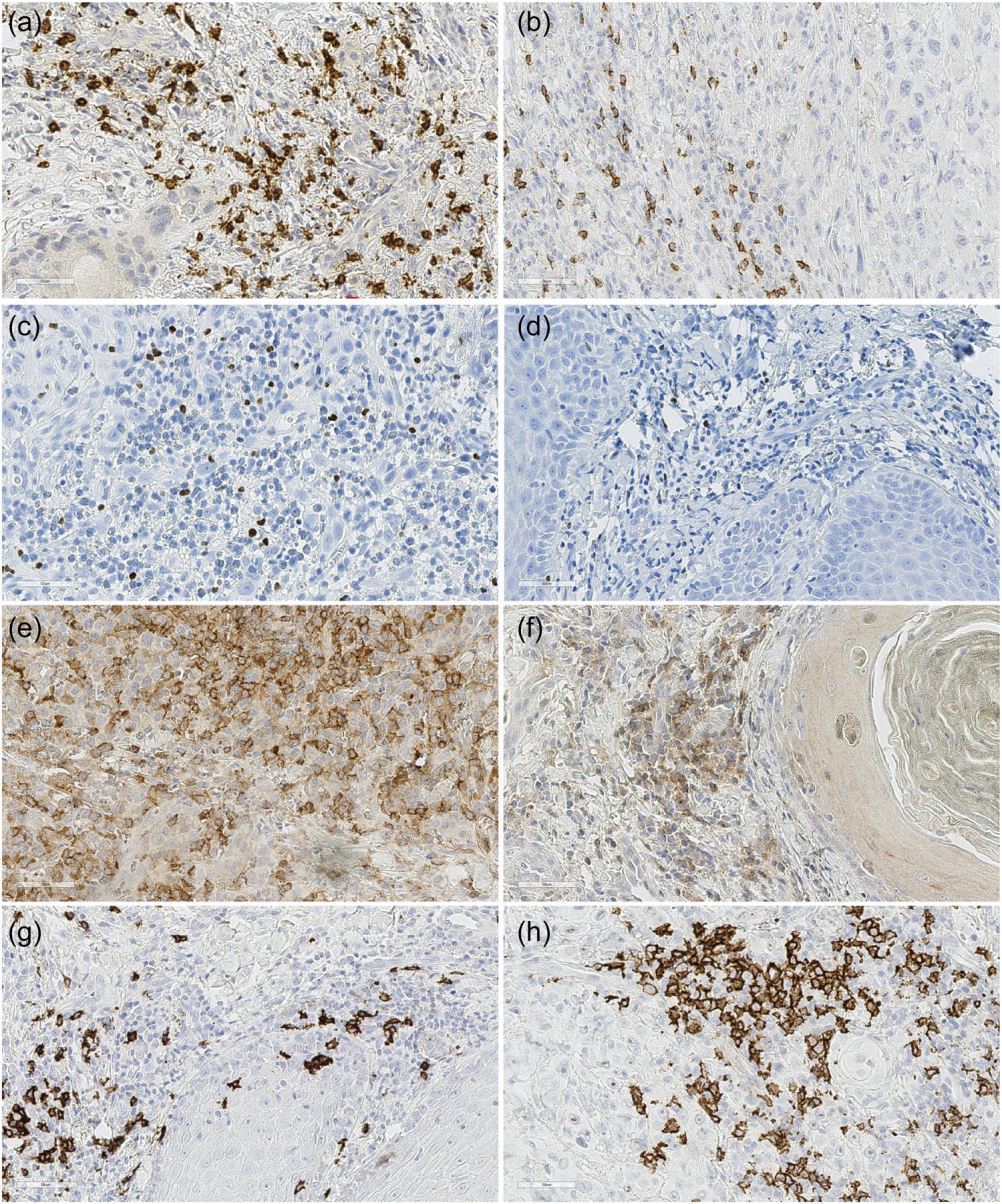
Representative images of immunohistochemical staining showing the presence of immune cell subsets at the invasive tumor front. (a) Visualization of the CD8^+^ cell infiltrates in a tumor with low tumor budding; (b) visualization of the CD8^+^ cell infiltrates in a tumor with high tumor budding; (c) visualization of the FoxP3^+^ cell infiltrates in a tumor from a toombak dipper; (d) visualization of the FoxP3^+^ cell infiltrates in a tumor from a non‐toombak user; (e) visualization of the CD4^+^ cell infiltrates in a poorly differentiated tumor; (f) visualization of the CD4^+^ cell infiltrates in a well‐differentiated tumor; (g) visualization of the CD20^+^ cell infiltrates in a Stage II tumor; and (h) visualization of the CD20^+^ cell infiltrates in a Stage IV tumor.

Increased CD8^+^ cytotoxic T‐cell infiltration in the stroma of ITF was found to be associated with low TB (phi = −0.50, *p* = .019) (Figure 3a,b). Further, toombak sites showed moderate correlation with FoxP3^+^ T‐regs infiltration (phi = 0.54, *p* = .020) (Figure 3c,d). Regarding intraepithelial tumoral infiltration, dense CD4^+^ cell infiltrate associated with poor tumor differentiation (phi = 0.45, *p* = .040) (Figure 3e,f), and higher CD4^+^ (phi = 0.46, *p* = .046) and CD20^+^ infiltration (phi = 0.69, *p* = .003) correlated with advanced tumor stage (Figure 3g,h).

### 3.6 Inflammatory biomarkers as prognostic indicators of OSCC

**Figure 4:**
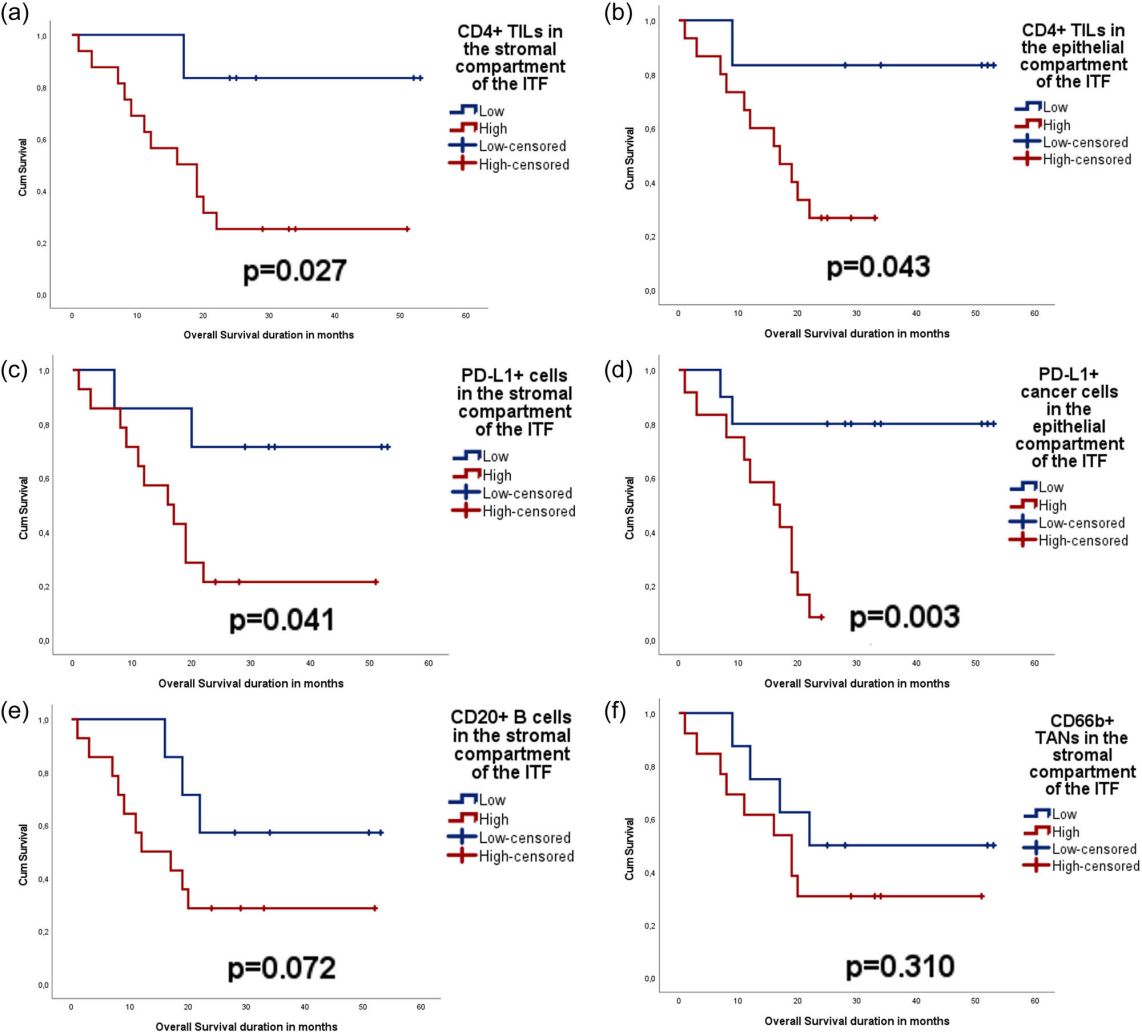
Kaplan–Meier curves showing distribution and survival analysis of the ITF of oral tumors (*n* = 22) according to (a) the amount of CD4^+^ cells infiltration into the stroma, (b) the amount of CD4^+^ cell infiltration into the tumor epithelium; (c) the amount of PD‐L1^+^ cell infiltration into the stroma; (d) the expression of PD‐L1 in the tumor epithelium; (E) the amount of B‐cell infiltration (CD20^+^ cells) into the stroma; and (f) the amount of neutrophils (CD66b^+^ cells) into the stroma. ITF, invasive tumor front; TIL, tumor‐infiltrating lymphocyte; TAN, tumor‐associated neutrophil.

Infiltration of CD4^+^ lymphocytes to stromal (*p* = .027) and intraepithelial compartment (*p* = .043) was associated with poor OS. Likewise, the presence of PD‐L1^+^ immune cell infiltrate in the stroma (*p* = .041) and expression of PD‐L1 in the tumor cells of the epithelial compartment (*p* = .003) were associated with poor OS. The same trend was observed for CD20^+^ (*p* = .072) and CD66b^+^ (*p* = .310) cell infiltration in the stroma, but the association was not statistically significant (Figure 4).

Furthermore, Cox regression analysis (age‐ and tumor stage‐adjusted) identified abundant PD‐L1^+^ tumor cells in the epithelial compartment as an independent indicator of increased hazard for poor OS (hazard ratio = 7.466, *p* = .018, overall *χ* ^2^ score = 12.01, *p* = .007) (Supporting Information: Table S5). CD4 in both compartments and PD‐L1 in the stromal compartment did not show any statistically significant findings when analyzed using the same model.

## 4 DISCUSSION

Although based on a relatively small cohort of OSCC patients from Sudan, the present study identifies intraepithelial PD‐L1 expression as an independent prognosticator of OS by using an age‐ and tumor‐stage‐adjusted Cox regression model. Several previous studies on various OSCC patient cohorts showed that high PD‐L1 expression correlated to metastasis, recurrence, poor DSS, and poor OS (Adamski et al., 2021; Lin et al., 2015; Naruse et al., 2020; de Vicente et al., 2019). This is in agreement with the independent prognostic value found by us in this study for PD‐L1. However, there are some other controversial findings which show that PD‐L1 expression could be an independent favorable prognostic factor for OS in OSCC (Ahn et al., 2017). The heterogeneity of findings and clinical correlations of PD‐L1 expression in various OSCC cohorts could be related to differences in the sociodemographical characteristics and risk factors associated with the various cohorts investigated. Given the fact that immunotherapy with PD‐L1 inhibitors is already introduced in clinics for the management of advanced OSCC in some countries and there is a desire to expand it to less advanced OSCC in more countries (Mohan et al., 2019), characterization and analysis of the prognostic impact of the presence of the immune check inhibitors are very important for OSCC treatment policy making and should be performed on various OSCC cohorts with different sociodemographic characteristics.

Of note, higher expression of both CD4 and PD‐L1 in both epithelial and stromal compartments at the ITF was found associated with poor OS in univariate analysis. Although we do not know the functionality of the cells that we have identified by the CD4 biomarker and their distribution into different CD4 subtypes, this might indicate that the high infiltration with CD4^+^ T‐helper cells and PD‐L1^+^ subsets at the ITF may contribute to a protumor immune host response. Supporting the view that CD4+ T‐helper cell infiltration might be an indicator of protumor response, is also the current finding that intra‐epithelial CD4+ infiltration at the ITF correlated with advanced tumor stage and poor differentiation. Nevertheless, this seems to be contrary to the literature that indicated a higher density of CD4^+^ TIME to be associated with less advanced disease and with favorable prognosis (Ahn et al., 2017; Badoual et al., 2006; Chen et al., 2018; Wolf et al., 2015). On the other hand, several studies that focused on head and neck cancer did not find the abundant CD4 infiltrates to be associated with clinical outcomes (Balermpas et al., 2014; Distel et al., 2009; Nordfors et al., 2013; Wansom et al., 2012). In line with our present results is another previous study on OSCC that describes an association between high CD4^+^ intratumoral cell counts and poor survival (Moreira et al., 2010).

Another important observation of the current study is that high stromal FoxP3^+^ infiltrates were associated with toombak dipping sites. Although the habit of toombak use was self‐reported, this might indicate an immunosuppressive host response in toombak‐related OSCC lesions. T‐regs are usually related to a suppressive host immune response; these cells were proven to impair proliferation, activation, and effector functions of several immune cell subsets, such as CD8^+^ and CD4^+^ T cells, and therefore were related to metastasis and disease progression in different cancer types (Dayan et al., 2012; Song et al., 2016; Weller et al., 2014; Zhang et al., 2019). The correlations we have identified between high FoxP3^+^ infiltrates and higher expression of epithelial PD‐L1 in OSCC tumors, together with the associations of high PD‐L1 expression with poor OS, suggest the involvement of FoxP3^+^ T‐regs in regulating PD‐l/PD‐L1 immune checkpoint and promotion of worse prognosis in OSCC. This is contradictory to a previous study on oropharyngeal SCC cases, which found that patients with low infiltration of T‐regs in the stroma at the ITF (“FoxP3‐excluded” tumors) had a poorer survival in comparison to patients with high stromal infiltration of T‐regs at the ITF (Yoshioka et al., 2008). Thus, this requires further investigation in larger cohorts of OSCC.

An interesting observation was that increased CD8^+^ lymphocyte infiltration was found to be associated with low TB, which might confirm the role of these cells in antitumor surveillance at the ITF. This corroborates well with previous findings from colorectal cancer, which found that a low CD8^+^ lymphocyte/TB index correlated to lymph node metastasis, invasion into vessels, and unfavorable clinical outcome in a multivariate analysis and this index was proposed as a forthcoming prognostic tool for colorectal cancer patients (Lugli et al., 2009).

Furthermore, we found that CD20^+^ intraepithelial infiltration at the ITF is associated with advanced tumor stage. This is contrary to our previous study in which CD20^+^ infiltration in the tumor‐associated stroma of TC was found associated with small‐sized tumors (Gaafar et al., 2022) and to previous literature showing high CD20^+^ B‐cell infiltration in TIME to correlate to favorable outcomes in OSCC (Ahn et al., 2017; Taghavi et al., 2018; Wirsing et al., 2018). These contradictions also indicate that further investigations are needed to elucidate the associations between B‐cell infiltrates with OSCC progression and outcomes to be performed in larger cohorts. Since most of the patients in this current cohort presented at Stage IV with large tumors, lymph node involvement, and WPI (Type 4), there is a need to validate the current results in cohorts with early OSCC stages.

## 5 CONCLUSIONS

This study showed that epithelial PD‐L1 expression at the ITF is an independent prognosticator for OSCC.

## AUTHOR CONTRIBUTIONS

*Study conceptualization*: Nuha M. Gaafar, Tarig Al‐Hadi Osman, Anne C. Johannessen, Elisabeth S. Nginamau, and Daniela‐Elena Costea. *Collection of patient material*: Nuha M. Gaafar, Israa Abdulrahman Ahmed, Mariam Elsheikh, and Ahmed M. Suleiman. *Experiments*: Nuha M. Gaafar, Harsh Dongre, and Siren Fromreide. *Data analyses*: Nuha M. Gaafar and Tarig Al‐Hadi Osman. *Preparation of figures and manuscript*: Nuha M. Gaafar. *Supervision*: Tarig Al‐Hadi Osman, Ahmed M. Suleiman, Anne C. Johannessen, Elisabeth S. Nginamau, and Daniela‐Elena Costea. All authors participated in writing and editing the manuscript with the approval of the submitted version.

## CONFLICT OF INTEREST

The authors declare no conflict of interest.

## Supporting information

Supporting information.

## Data Availability

Data are available on request from the authors.
