# Supplementary material for: Epithelial PD‐L1 expression at tumor front predicts overall survival in a cohort of oral squamous cell carcinomas from Sudan
Source: Clin Exp Dent Res. 2022 Sep 30;8(6):1467–77. doi: 10.1002/cre2.666 (PMC9760153; doi:10.1002/cre2.666)
Supplement: Supplementary file 1 — Supporting information. [file CRE2-8-1467-s001.docx]

**Supplementary Data**

**Table S1**: Clinical parameters: age, missed teeth status (MT), decayed teeth status (DT) and periodontal treatment needs (community periodontal index of treatment needs - CPITN); as predictors of overall survival using univariate and multivariate regression model.

| Parameter | Number | Univariate analysis | | Multivariate analysis | |  |
| --- | --- | --- | --- | --- | --- | --- |
|  |  | *P* value | HR (CI 95%) | *P* value | HR (CI 95%) | |
| Age | 22 | 0.021 | 1.066 (1.010-1.127) | 0.037 | 1.145 (1.008-1.300) | |
| MT | 22 | 0.044 | 1.062 (1.002-1.126) | 0.200 | 0.919 (0.808-1.046) | |
| DT | 22 | 0.026 | 0.752 (0.585-0.967) | 0.010 | 0.578 (0.380-0.880) | |
| CPTIN | 22 | 0.077 | 1.957 (0.929-4.120) | 0.062 | 2.421 (0.955-6.135) | |

**Table S2** Expression of different immune biomarkers in stromal and epithelial tumor compartments evaluated at the invasive tumour front of oral squamous cell carcinoma.

| Marker | Stroma | | Epithelium | | *P* value | Direction |
| --- | --- | --- | --- | --- | --- | --- |
|  | **Mean** | **SD** | **Mean** | **SD** |  |  |
| CD4 | 11203.39 | 3337.30 | 5557.53 | 3123.93 | < 0.001 | Stroma > epi |
| CD8 | 65.59 | 15.30 | 53.41 | 17.39 | < 0.001 | Stroma > epi |
| FoxP3 | 434.52 | 362.47 | 124.81 | 116.68 | < 0.001 | Stroma > epi |
| CD20 | 7274.29 | 3102.97 | 797.73 | 1021.44 | < 0.001 | Stroma > epi |
| CD66b | 52.53 | 11.57 | 43.86 | 11.84 | 0.001 | Stroma > epi |
| PD-L1 | 400.51 | 693.71 | 1030.76 | 1963.82 | 0.502 | Epi > stroma |
| M1 | 0.86 | 1.33 | - | - | - | - |
| M2 | 2.04 | 1.93 | - | - | - | - |
| M1:M2 | 0.90 | 1.77 | - | - | - | - |

**Table S3** Correlations between various immune biomarkers in the stromal compartment at the invasive tumour front of oral squamous cell carcinoma.

| Marker | CD4 | | | CD8 | | | FoxP3 | | | CD20 | | | CD66b | | |
| --- | --- | --- | --- | --- | --- | --- | --- | --- | --- | --- | --- | --- | --- | --- | --- |
|  | χ² | Phi coefficient | *P* | χ² | Phi coefficient | *P* | χ² | Phi coefficient | *P* | χ² | Phi coefficient | *P* | χ² | Phi coefficient | *P* |
| CD4 |  | | | 14.44 | 0.81 | < 0.001 | 0.03 | -0.04 | 0.856 | 0.13 | 0.08 | 0.717 | 10.66 | 0.71 | 0.001 |
| CD8 | 14.44 | 0.81 | < 0.001 |  | | | 0.01 | 0.02 | 0.933 | 0.11 | -0.07 | 0.743 | 10.10 | 0.69 | 0.001 |
| FoxP3 | 0.03 | -0.04 | 0.856 | 0.01 | 0.02 | 0.933 |  | | | 2.68 | 0.36 | 0.102 | 0.94 | -0.21 | 0.332 |
| CD20 | 0.13 | 0.08 | 0.717 | 0.11 | -0.07 | 0.743 | 2.68 | 0.36 | 0.102 |  | | | 0.29 | 0.12 | 0.589 |
| CD66b | 10.66 | 0.71 | 0.001 | 10.10 | 0.69 | 0.001 | 0.94 | -0.21 | 0.332 | 0.29 | 0.12 | 0.589 |  | | |
| PD-L1 | 0.13 | 0.08 | 0.717 | 0.11 | -0.07 | 0.743 | 0.11 | -0.07 | 0.743 | 0.11 | -0.07 | 0.743 | 0.20 | -0.10 | 0.658 |
| M1/M | 0.13 | 0.36 | 0.550 | 0.56 | 0.17 | 0.456 | 0.36 | 0.13 | 0.550 | 0.89 | 0.21 | 0.345 | 0.12 | -0.08 | 0.729 |
| M2/M | 0.73 | -0.19 | 0.394 | 1.94 | -0.31 | 0.163 | 0.05 | 0.05 | 0.831 | 0.22 | 0.11 | 0.637 | 2.33 | -0.35 | 0.127 |
| M1:M2 | 2.54 | 0.36 | 0.111 | 2.81 | 0.38 | 0.094 | 0.36 | 0.13 | 0.550 | 1.56 | 0.29 | 0.211 | 0.35 | 0.14 | 0.552 |
| Marker | PD-L1 | | | M1/M | | | M2/M | | | M1:M2 | | |  |  |  |
|  | χ² | Phi coefficient | *P* | χ² | Phi coefficient | *P* | χ² | Phi coefficient | *P* | χ² | Phi coefficient | *P* |  |  |  |
| CD4 | 0.13 | 0.08 | 0.717 | 0.36 | 0.13 | 0.550 | 0.73 | -0.19 | 0.394 | 2.54 | 0.36 | 0.111 |  |  |  |
| CD8 | 0.11 | -0.07 | 0.743 | 0.56 | 0.17 | 0.456 | 1.94 | -0.31 | 0.163 | 2.81 | 0.38 | 0.094 |  |  |  |
| FoxP3 | 0.11 | -0.07 | 0.743 | 0.36 | 0.13 | 0.550 | 0.05 | 0.05 | 0.831 | 0.36 | 0.13 | 0.550 |  |  |  |
| CD20 | 0.11 | -0.07 | 0.743 | 0.89 | 0.21 | 0.345 | 0.22 | 0.11 | 0.637 | 1.56 | 0.29 | 0.211 |  |  |  |
| CD66b | 0.20 | -0.10 | 0.658 | 0.12 | -0.08 | 0.729 | 2.33 | -0.35 | 0.127 | 0.35 | 0.14 | 0.552 |  |  |  |
| PD-L1 |  | | | 0.28 | -0.12 | 0.599 | 5.00 | 0.51 | 0.025 | 0.05 | -0.05 | 0.829 |  |  |  |
| M1/M | 0.28 | -0.12 | 0.599 |  | | | 2.54 | 0.36 | 0.111 | 2.81 | 0.38 | 0.094 |  |  |  |
| M2/M | 5.00 | 0.51 | 0.025 | 2.54 | 0.36 | 0.111 |  | | | 1.94 | -0.31 | 0.163 |  |  |  |
| M1:M2 | 0.05 | -0.05 | 0.829 | 2.81 | 0.38 | 0.094 | 1.94 | -0.31 | 0.163 |  | | |  |  |  |

**Table S4** Correlations between various immune biomarkers in the epithelial compartment at the invasive tumour front of oral squamous cell carcinoma.

| Marker | CD4 | | | CD8 | | | FoxP3 | | | CD20 | | | CD66b | | | PD-L1 | | |
| --- | --- | --- | --- | --- | --- | --- | --- | --- | --- | --- | --- | --- | --- | --- | --- | --- | --- | --- |
|  | χ² | Phi coefficient | *P* | χ² | Phi coefficient | *P* | χ² | Phi coefficient | *P* | χ² | Phi coefficient | *P* | χ² | Phi coefficient | *P* | χ² | Phi coefficient | *P* |
| CD4 |  | | | 0.01 | 0.02 | 0.919 | 2.91 | 0.37 | 0.088 | 7.64 | 0.60 | 0.006 | 0.02 | 0.03 | 0.890 | 9.24 | 0.66 | 0.002 |
| CD8 | 0.01 | 0.02 | 0.919 |  | | | 0.04 | -0.04 | 0.848 | 2.65 | 0.36 | 0.104 | 6.39 | 0.55 | 0.011 | 0.03 | -0.04 | 0.864 |
| FoxP3 | 2.91 | 0.37 | 0.088 | 0.04 | -0.04 | 0.848 |  | | | 2.65 | 0.36 | 0.104 | 6.39 | -0.55 | 0.011 | 3.88 | 0.43 | 0.049 |
| CD20 | 7.64 | 0.60 | 0.006 | 2.65 | 0.36 | 0.104 | 2.65 | 0.36 | 0.104 |  | | | 0.18 | 0.09 | 0.670 | 2.93 | 0.37 | 0.087 |
| CD66b | 0.02 | 0.03 | 0.890 | 6.39 | 0.55 | 0.011 | 6.39 | -0.55 | 0.011 | 0.18 | 0.09 | 0.670 |  | | | 0.73 | -0.18 | 0.392 |
| PD-L1 | 9.24 | 0.66 | 0.002 | 0.03 | -0.04 | 0.864 | 3.88 | 0.43 | 0.049 | 2.93 | 0.37 | 0.087 | 0.73 | -0.18 | 0.392 |  | | |

**Table S5** Cox regression model for prognostication of overall survival in oral squamous cell carcinoma.

| Marker | Number | *p* value | HR | CI (95%) |
| --- | --- | --- | --- | --- |
| PD-L1 | 22 | 0.018 | 7.466 | (1.408-39.584) |
| Age | 22 | 0.026 | 4.985 | (1.213-20.492) |
| Tumour stage | 22 | 0.328 | 1.552 | (0.643-3.746) |
